# Evaluation of Neurofilament Light Chain as a Biomarker of Neurodegeneration in X-Linked Childhood Cerebral Adrenoleukodystrophy

**DOI:** 10.3390/cells11050913

**Published:** 2022-03-07

**Authors:** Hongge Wang, Matthew D. Davison, Martin L. Kramer, Weiliang Qiu, Tatiana Gladysheva, Ruby M. S. Chiang, Can Kayatekin, David R. Nascene, Leyla A. Taghizadeh, Carina J. King, Erin E. Nolan, Ashish O. Gupta, Paul J. Orchard, Troy C. Lund

**Affiliations:** 1 Translational Sciences, Sanofi Research, Sanofi, Framingham, MA 01701, USA; hw8y@yahoo.com (H.W.); matthewdavison14@gmail.com (M.D.D.); Martinkramer4@gmail.com (M.L.K.); 2 Nonclinical Efficacy and Safety, Department of Biostatistics and Programming, Sanofi Development, Sanofi, Framingham, MA 01701, USA; Weiliang.Qiu@sanofi.com; 3 Integrated Drug Discovery, Sanofi Research, Sanofi, Waltham, MA 02451, USA; Tatiana.Gladysheva@sanofi.com; 4 Rare and Neurological Diseases Research Therapeutic Area, Sanofi, 49 New York Avenue, Framingham, MA 01701, USA; Ruby.Vashisth@sanofi.com (R.M.S.C.); Can.Kayatekin@sanofi.com (C.K.); 5 Department of Diagnostic Radiology, University of Minnesota Medical Center, Minneapolis, MN 55455, USA; nasc0001@umn.edu; 6 Division of Pediatric Blood and Marrow Transplantation, University of Minnesota, Minneapolis, MN 55455, USA; taghi012@umn.edu (L.A.T.); king1051@umn.edu (C.J.K.); enolan@umn.edu (E.E.N.); gupta461@umn.edu (A.O.G.); orch001@umn.edu (P.J.O.)

**Keywords:** adrenoleukodystrophy, biomarkers, neurofilament light chain

## Abstract

Cerebral adrenoleukodystrophy (CALD) is a devastating, demyelinating neuroinflammatory manifestation found in up to 40% of young males with an inherited mutation in *ABCD1*, the causative gene in adrenoleukodystrophy. The search for biomarkers which correlate to CALD disease burden and respond to intervention has long been sought after. We used the Olink Proximity Extension Assay (Uppsala, Sweden) to explore the cerebral spinal fluid (CSF) of young males with CALD followed by correlative analysis with plasma. Using the Target 96 Neuro Exploratory panel, we found that, of the five proteins significantly increased in CSF, only neurofilament light chain (NfL) showed a significant correlation between CSF and plasma levels. Young males with CALD had a 11.3-fold increase in plasma NfL compared with controls. Importantly, 9 of 11 young males with CALD who underwent HCT showed a mean decrease in plasma NfL of 50% at 1 year after HCT compared with pre-HCT levels. In conclusion, plasma NfL could be a great value in determining outcomes in CALD and should be scrutinized in future studies in patients prior to CALD development and after therapeutic intervention.

## 1. Introduction

Genetic mutation in the *ABCD1* gene leads to X-ALD, affecting very long chain fatty acid (VLCFA) processing (and resultant VLCFA buildup), which can manifest as several phenotypes, including adrenal insufficiency, adrenomyeloneuropathy (AMN), or a devasting demyelinating cerebral form, CALD, which occurs in up to 40% of young males with ALD before the age of 18 years [1]. The onset of cerebral disease is defined by demyelinating lesions seen on magnetic resonance imaging (MRI) which are surrounded by a “garland ring” of gadolinium enhancement [2]. Following onset, CALD is usually progressive and results in death within a decade [3]. The only accepted medical treatment is hematopoietic cell transplantation (HCT), and upon successful engraftment of donor-derived cells, the gadolinium enhancement resolves, and the cerebral process may be arrested [3,4,5]. Ex vivo autologous gene therapy is also being explored with promising results in early clinical trials [6].

Triggers or predictors of CALD remain elusive, though several groups have reported biomarkers that are correlative with the extent disease. For example, an imaging biomarker measured the extent of cerebral disease involvement that can be assessed on MRI by a scoring system developed by Loes et al., which enumerates the brain structures/areas affected [7]. The Loes MRI severity score stratification also allows establishment of risk groups based on this score and has been associated with outcomes after HCT [5]. Additionally, we have previously described that the measurement of the volume of gadolinium enhancement correlated to the timing of the ultimate resolution of the gadolinium signal after HCT and is also inversely related to neurologic progression after HCT [8].

In search of blood (plasma/serum)-based or cerebral spinal fluid (CSF)-based biomarkers, we previously have shown that CSF IL-8, MCP-1, and MIP-1b, MMP2, MMP9, MMP10, and TIMP1 were significantly elevated in young males with CALD [9,10]. Plasma SDF-1 levels have also been shown to correlate to MRI Loes score [10]. Finally, both CSF and plasma chitotriosidase have been associated with the presence of CALD and correlate to MRI Loes scores, as well neurologic progression after HCT [11].

Recently, neurofilament light chain protein (NfL) has been explored as a potential blood biomarker for monitoring neurodegeneration in patients with a variety of neurologic diseases including CALD [12]. NfL is a structural element of neurons and released into the cerebral spinal fluid (CSF) and blood after neuronal damage [13]. In AMN, Weinhoffer et al. found elevated NfL levels correlating with a greater degree of myelopathy-related disability. NfL was a predictor to differentiate those who later convert to CALD versus those that did not convert [12]. While most of their data was in adults, they also studied 13 young males with cerebral ALD (CALD) including 5 young males post-HCT and found that elevated NfL levels were associated with CALD, which abated after HCT [12]. Here, we report our findings exploring plasma NfL in 26 pediatric patients with CALD with 12 patients having NfL measured 1 year after successful HCT.

## 2. Materials and Methods

CALD plasma and CSF were obtained at initial consultation visit and disease assessment in the University of Minnesota ALD Comprehensive Clinic. Pediatric control plasma was purchased from DxBiosamples (San Diego, CA, USA) and from Fidelis (Sofia, Bulgaria). Control adult CSF samples were obtained from BioIVT, (Westbury, New York, NY, USA). Adult control patients were considered “healthy” if they were in a state of general wellness, have no underlying or chronic conditions, and were not currently suffering from an infectious disease. NfL was measured in aliquots of the plasma and CSF samples using the Olink immune PCR assay (Uppsala, Sweden) with the Neurology Exploratory Panel (92 proteins), which includes NfL, and using the manufacturers recommended protocols [14,15]. Specifically, proximity extension assay (PEA) technology used for the Olink protocol has been well described and enables 92 analytes to be analyzed simultaneously, using 1 µL of each sample [14]. In brief, pairs of oligonucleotide-labeled antibody probes bind to their targeted protein, and if the two probes are brought in close proximity the oligonucleotides will hybridize in a pair-wise manner. The addition of a DNA polymerase leads to a proximity-dependent DNA polymerization event, generating a unique PCR target sequence. The resulting DNA sequence was subsequently detected and quantified using a microfluidic real-time PCR instrument (Biomark HD, Fluidigm, South San Francisco, CA, USA) and then was quality controlled and normalized using an internal extension control and an inter-plate control, to adjust for intra- and inter-run variation. The final assay read-out is presented in normalized protein expression (NPX) values, which is an arbitrary unit on a log2-scale where a high value corresponds to a higher protein expression. All assay validation data (detection limits, intra- and inter-assay precision data, etc.) are available on the manufacturer’s website (www.olink.com, accessed on 3 February 2022). The resultant assay normalized protein expression (NPX) values are given in log_2_. Anti-log_2_ transformation of the estimated difference and its 95% CI provide fold-change and its 95% CI. Data were analyzed using GraphPad Prism v7.03 (GraphPad, San Diego, CA, USA) and JMP v16.0 (University of Illinois, Urbana-Champaign, IL, USA).

## 3. Results

We measured CSF biomarker levels by proximity extension assay (Olink, Uppsala, Sweden) in a cohort of young males with CALD (*n* = 11, Table 1) evaluated in the ALD Clinic at the University of Minnesota. Patients over 18 years of age were excluded. Patients had a wide variety of cerebral involvement based on MRI Loes score (median = 8.3).

**Figure 1:**
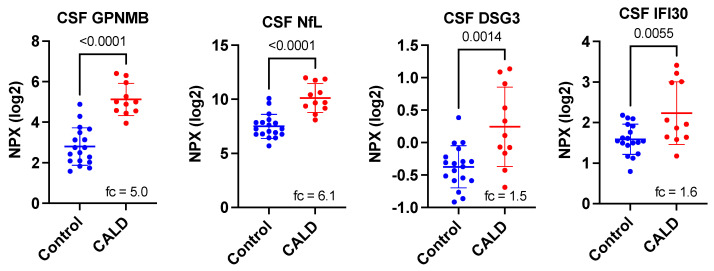
Significantly elevated CSF proteins between CALD and control patients identified on the Olink Neurology Exploratory Panel. Fc indicated fold-change after conversion to linear scale. *p*-values are derived from a Student’s *t*-test.

Assessment of initial results comparing CALD CSF with that from individuals without CALD (note only control adult CSF was available), we found 28 of the 92 proteins on the Neurology Exploratory Panel to show significant differences (either increased or decreased shown in Table 2). Our initial search for disease correlative biomarkers focused on the 4 proteins (GPNMB, NEFL, DSG3, and IFI30) that were elevated in CALD CSF, though there were 24 proteins lower in CALD CSF. The proteins GPNMB, NEFL (also termed NfL), DSG3, and IFI30 were elevated 5.0-, 6.1-, 1.5-, and 1.6-fold, respectively, in the CSF of young males with CALD (Figure 1). With Bonferroni correction, only two proteins, GPNMB and NfL, were highly significant.

Interestingly, of these five highly elevated proteins (Figure 1), only GPNMB and NfL in CSF have previously been shown as biomarkers in neurological disease [16,17,18].

**Figure 2:**
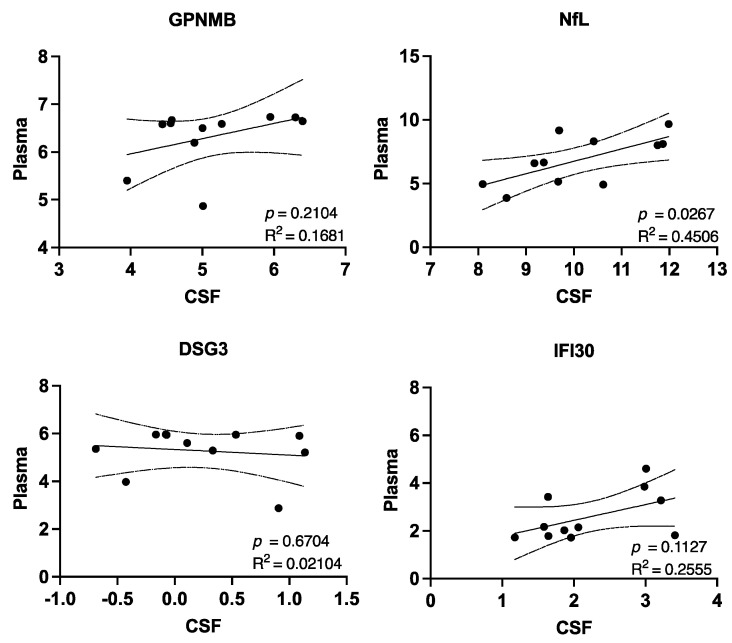
Correlation between CSF protein and plasma protein NPX values determined from Olink Neurology Exploratory Panel. R^2^ and *p*-values were derived from a simple linear regression. Dotted lines indicate the 95% confidence intervals.

Given that obtaining CSF from a patient requires a sedated procedure with a risk of a “spinal headache”, a plasma-related biomarker is more desirable. Evaluation of the five previously identified protein’s CSF concentration compared with their matched plasma concentration indicated that only NfL significantly correlated with plasma levels (Figure 2).

**Figure 3:**
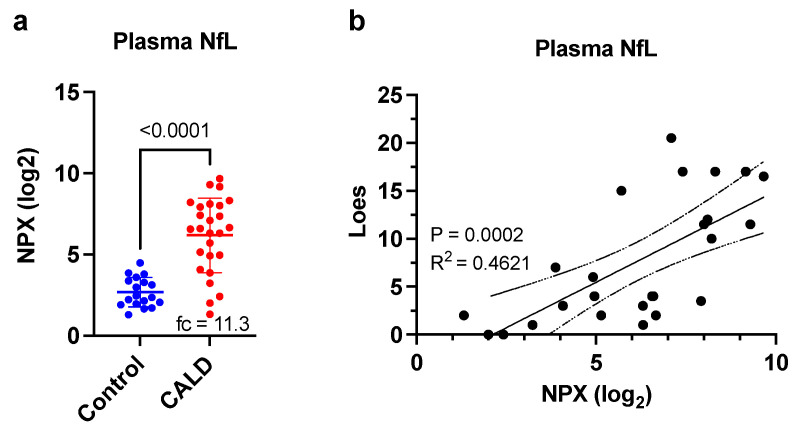
Elevated plasma NfL in CALD patients correlated with cerebral disease level. (**a**) Comparison of plasma NfL levels between CALD patients and a pediatric control group. NPX values determined from Olink Neurology Exploratory Panel. Fc indicated fold-change after conversion to linear scale. *p*-values are derived from a Student’s *t*-test. (**b**) Correlation between Loes score and plasma NfL. R^2^ and *p*-values were derived from a simple linear regression. Dotted lines indicate the 95% confidence intervals.

We compared NfL plasma levels from a larger set of CALD patients to a cohort of pediatric control plasma samples and found that NfL was increased 11.3-fold in CALD patients (Figure 3a, *p* < 0.0001). We next assessed for a correlation between plasma NfL and MRI Loes score and found a very good association between them (Figure 3b, *p* = 0.0002, R^2^ = 0.4621).

**Figure 4:**
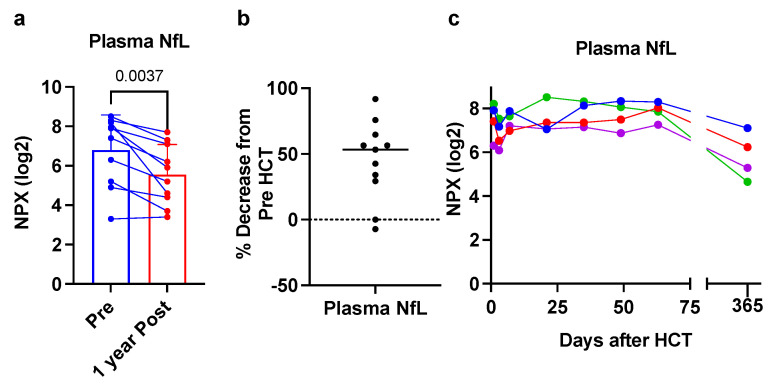
Response of plasma NfL to HCT. (**a**) Eleven paired samples before and 1 year after HCT. *p*-value from a Student’s paired *t*-test. (**b**) Decreased plasma NfL. NPX value were converted to linear scale prior to calculating percent decrease. (**c**) Frequent longitudinal plasma samples in four CALD patients after HCT to 365 days post-HCT (Pre-HCT is timepoint 0). Each color represents a unique patient.

Eleven patients were assessed pre- and post-HCT. Figure 4a,b shows that 9 of the 11 CALD patients showed decreased plasma NfL levels at 1 year post-HCT, with an overall mean decrease of 50%. Finally, 4 of the patients we assessed had more detailed serial sampling of plasma after HCT. Figure 4c shows that plasma NfL levels were stable in close approximation to HCT (to 60 days post-HCT), but by 1 year after HCT, levels were decreased from pre-HCT levels.

## 4. Discussion

Our study involved a comprehensive assessment of CALD CSF for potential biomarkers. We found a clear increase in CSF and plasma NfL levels in a large cohort of young males with CALD. Furthermore, we find a good correlation between levels of NfL and the amount of cerebral involvement as seen on MRI. Finally, plasma NfL showed lower levels after successful bone marrow transplant in the majority of young males (9 of 11).

Neurofilaments are structural elements of neuronal axons. Many pathologic processes in the brain involve the destruction of neurons (to some extent) releasing neurofilaments into the CSF and circulation [19]. Until recently, only CSF could be used to detect NfL due to the very low levels of NfL in plasma. With the recent development of ultrasensitive detection systems such as SiMoA and Olink, plasma can be reliably assessed for NfL [19]. Investigations into NfL as a biomarker have been wide-ranging, from head injury to stroke and, of course, to neurological disease [18,20,21].

In addition to injury and disease, age related changes in serum NfL have been reported [22,23,24]. For example, Khalil et al. reported that individuals greater than 60 years of age showed consistently higher serum NfL levels, which were associated with brain volume loss and white matter hyperintensity volume [22]. On the other end of the age spectrum, Nitz et al. evaluated pediatric plasma NfL levels in control patients and found higher concentrations of NfL in patients less than 4 years old (a median of 7.12 pg/mL) compared with those of patients 5–18 years of age (a median of 4.07 pg/mL, *p* = 0.004 for comparison between the groups) [23]. There was no difference between sexes nor amongst older children up to age 18. Taken into context with our study, the young males we studied were clearly lower in age than 60 years with a median of 6.9 years (range 4–15.5 years), which is also within a “stable NfL” range of from Nitz’s study population. Where these age-related differences may play a role is in the area of newborn screening for ALD and trying to utilize biomarkers in very young children to predict those that might be predisposed to developing cerebral disease. Certainly, and any age-related differences in biomarker concentrations would have to be considered.

Weinhofer et al. recently showed that young males with childhood CALD (*n* = 13) or male adults with CALD (*n* = 11) had significantly elevated plasma/serum NfL levels compared with controls (79.5-fold and 8.6-fold, respectively), as well as a significant positive correlation between plasma/serum NfL levels and the Loes MRI severity score (R^2^ = 0.73, *p* = 0.002) [12], both of which are in agreement with our findings shown here. Our increased plasma NfL was not as high at their report (at 11.3 fold), but might be explained by a difference in technologies used to measure NfL (SiMoA versus Olink). In addition, they showed that, for CALD patients that underwent HCT, plasma/serum NfL decreased after HCT (median time was 5.1 years post-HCT). The time course of NfL reduction following HCT was not further characterized, but longitudinal plasma NfL levels shown for two patients with CALD before and after HCT suggested that decreases in NfL would not be apparent until 1 year post-HCT [12]. Our data in post-HCT patients is in overall good agreement with this finding as well. Initially, we did not observe any real decrease in NfL (first 60 days) post-HCT and some levels appeared slightly increased. This may be a result of neurotoxicity from the chemotherapy used in conditioning the patients prior to HCT, which is known to occur. In addition, successful engraftment of the brain with donor cells may occur over weeks to months after HCT allowing for a prolonged course before NfL levels stabilize or fall. Of note, two patients in our cohort showed no real decrease in NfL level 1 year post-HCT but this did not correlate to any unusual or rapid advancement in disease symptoms or MRI findings (not shown). Overall, the aggregate NfL data do show a response to HCT. It is encouraging to show a biomarker responsive to therapy, which has been lacking in the field.

While our results and the work by Weinhoffer et al. focused on CALD, the use of NfL has been previously demonstrated in a wide variety of conditions, including amyotrophic lateral sclerosis (ALS), multiple sclerosis (MS), and most recently in COVID-19 infection [18,25,26]. In ALS, while it is not entirely clear that NfL can predict disease onset, there is evidence that high levels of Nfl predict a more rapid and aggressive course of disease [19]. In MS, NfL levels correlate with worse MRI findings, brain and spinal cord atrophy, clinical progression, and relapse [27,28]. In COVID-19 patients, rising longitudinal NfL measurements may identify patients at risk of mortality [25].

Finding elevated levels of a given biomarker associated with an increase in severity of disease is convenient; although we did find a number of CSF markers decreased in CALD patients, evidence to decipher their role is more challenging to come by, but there are a couple of examples worth mentioning [29,30,31,32]. We found CSF FUT8 levels decreased in CALD patients. FUT8-knockout mice show higher levels of Iba-1 and GFAP staining cells in lipopolysaccharide (LPS)-stimulated (conditional) models [29]. IL-6 also promoted greater levels of STAT3 signaling in FUT8-knockout primary astrocytes suggesting that FUT8 has a negative role in mediating inflammatory signals [29]. Another CSF protein decreased in CALD from our protein panel was CRIP2. CRIP2-knockout mice have been shown to have increased nociceptive behavior in models of inflammatory hyperalgesia compared with wild-type mice, suggesting an inhibitory role for this protein in generation of inflammatory pain [30]. Finally, CSF NXPH1 was roughly 20% of control levels in our study. Inhibition of NXPH1 (by specific micro-RNA expression) inhibits the LPS-induced proliferation, invasion, and inflammatory activation of astrocytes, suggesting its role in the inflammatory cascade; although, in this case, the loss of a proinflammatory mediator is a more difficult scenario to translate clinically and directly show cause and effect [32].

Our prior work to determine robust CALD biomarkers largely focused on CSF as a matrix, which some would argue is more invasive to obtain and often requires sedation for young children. Having a plasma-based biomarker is a useful advantage. Additionally, very few biomarkers in CALD have responded to treatment (often HCT), again allowing NfL to have more potential than prior biomarkers in this regard. While the full potential of NfL remains uncertain as a single biomarker, future studies will likely focus on combinations of biomarkers to predict clinical course pre- and post-HCT.

More importantly, there has been no robust marker to predict which ALD patients will develop CALD. We previously showed that antibodies to Profilin can be found with conversion to CALD [33], but this was limited in scope with only a handful of patients. The discovery biomarkers which can predate (or predict) CALD would be of upmost importance and allow physicians to deliver therapeutic measures very early in disease course.

## Figures and Tables

**Table 1:** Demographics of samples analyzed in this study.

Group	CSF CALD Baseline	CSF Control (Adult)	Plasma CALD Baseline	Plasma Control (Pediatric)
*n*	11	18	26	18
Age, years	8.4 (4.5–15.5)	43 (23–68)	6.9 (4–15.5)	7 (4–16)
Sex	Male	Male	Male	Male
Loes Score	8.3 (2–17)	NA	4 (0–20.5)	NA
NfL, NPX (IQR) ^1^	9.7 (9.2–12.0)	7.5 (6.8–7.9)	6.6 (4.7–8.3)	2.4 (1.9–3.4)

^1^ NPX (normalized protein expression) values are shown in log_2_ and IQR represents the interquartile range.

**Table 2:** Most significantly changed CSF proteins between CALD and control patients identified on the Olink Neurology Exploratory Panel.

Protein Symbol	Uniprot ID	Name	*p*-Value ^1^	Corrected *p*-Value ^2^	Fold-Change ^3^
GPNMB	Q14956	transmembrane glycoprotein NMB	2.01 × 10^−7^	1.85 × 10^−5^	5.00
NXPH1	P58417	neurexophilin 1	1.11 × 10^−6^	0.0001	−4.55
NEFL	P07196	neurofilament light chain	4.74 × 10^−6^	0.0004	6.13
CRIP2	P52943	Cysteine-rich protein 2	8.13 × 10^−6^	0.0007	−2.94
TDGF1	P13385	teratocarcinoma-derived growth factor 1	2.97 × 10^−5^	0.0027	−4.55
FUT8	Q9BYC5	fucosyltransferase 8	8.28 × 10^−5^	0.0076	−1.49
NPM1	P06748	nucleophosmin 1	0.0001	0.0092	−3.03
SMOC1	Q9H4F8	SPARC Related Modular Calcium Binding 1	0.0004	0.0368	−1.75
ADAM15	Q13444	disintegrin and metalloproteinase domain-containing protein 15	0.0009	0.0828	−1.92
DSG3	P32926	desmoglein-3	0.0014	0.1288	1.54
TBCB	Q99426	tubulin-folding cofactor B	0.0018	0.1656	−1.45
PLA2G10	O15496	group 10 secretory phospholipase A2 preproprotein	0.0025	0.2300	−1.75
LTBP3	Q9NS15	latent-transforming growth factor beta-binding protein 3	0.0027	0.2484	−1.20
AKT1S1	Q96B36	proline-rich AKT1 substrate 1	0.0027	0.2484	−1.19
IFI30	P13284	Gamma-interferon-inducible lysosomal thiol reductase	0.0055	0.5060	1.56
FKBP7	Q9Y680	peptidyl-prolyl cis-trans isomerase FKBP7	0.0059	0.5888	−1.22
TNFRSF13C	Q96RJ3	tumor necrosis factor receptor superfamily member 13C	0.0064	0.6624	−1.52
GGT5	P36269	glutathione hydrolase 5 proenzyme	0.0072	0.7084	−1.39
FGFR2	P21802	fibroblast growth factor receptor 2	0.0077	0.9384	−1.45
EIF4B	P23588	eukaryotic translation initiation factor 4B	0.0102	1.0000	−1.64
EPHA10	Q5JZY3	ephrin type-A receptor 10	0.0126	1.0000	−2.17
PRTFDC1	Q9NRG1	phosphoribosyltransferase domain-containing protein 1	0.0168	1.0000	−1.52
IL3RA	P26951	interleukin-3 receptor subunit alpha	0.0172	1.0000	−1.12
IL15	P40933	interleukin-15	0.0193	1.0000	−1.27
PHOSPHO1	Q8TCT1	phosphoethanolamine/phosphocholine phosphatase	0.0194	1.0000	−1.30
SRP14	P37108	signal recognition particle 14 kDa protein	0.0196	1.0000	−1.27
ASGR1	P07306	asialoglycoprotein receptor 1	0.0427	1.0000	−1.09
WWP2	O00308	NEDD4-like E3 ubiquitin-protein ligase WWP2	0.0481	1.0000	−1.22

^1^*p*-values derived from Student’s *t*-test. Shown are the proteins that were significantly different (*p* < 0.05). ^2^ Bonferroni correction applied. ^3^ Fold-change is CALD: control with fold-change, calculated after conversion to linear scale.

## Data Availability

The data presented in this study are available on request from the corresponding author.
